# Non-resectable pulmonary alveolar echinococcosis with multi-stage vertebral location

**DOI:** 10.1016/j.rmcr.2023.101886

**Published:** 2023-06-11

**Authors:** N. Belloumi, C. Habouria, S. Fidha, I. Bachouch, F. Chermiti Ben Abdallah, S. Fenniche

**Affiliations:** a Pulmonology department Pavilion 4, Abderrahmen Mami hospital, Ariana, Tunisia; b Faculty of medicine of Tunis, University of Tunis el manar, Tunisia

**Keywords:** Spine, Hydatidosis, Surgical treatment, Albendazole, Recurrence

## Abstract

Alveolar echinococcosis (also known as Hydatid cyst or Hydatydosis) is a zoonosis with a high degree of disability and morbidity. Bone echinococcosis is a rare presentation. Authors are always defending a personalized approach taking account of the particularities of the cyst location. Recognition of this syndrome is crucial because advances in medical and surgical management strategies have controlled and relieved symptoms in numerous cases. We report, hereby, a case of a patient with a thoracic spine alveolar echinococcosis of an unusual extension. We discussed the outcome of the treatment after fifteen years of follow-up.

## 1 Introduction

Alveolar echinococcosis (also known as Hydatid cyst or Hydatydosis) is caused by various species of tapeworms belonging to the genus Echinococcus. The most common among them is Echinococcus Granulosus [1]. It is an infrequent zoonosis with a high degree of disability and morbidity. The diagnosis is complicated by extended incubation time, diverse clinical manifestations, and mimicking of differential diagnoses. The primary organ affected is the liver (55–75%) followed by the lungs (15–35%) [2]. Other visceral localizations are possible, with vertebral involvement in only a few cases described worldwide [[3], [4], [5]]. Although vertebral alveolar echinococcosis seems to be rare, it might be under-diagnosed, and it might be seen more often as the number of people with immunocompromised conditions increases. Recognition of this syndrome is crucial because advances in medical and surgical management strategies have controlled and relieved symptoms in numerous cases. In addition, bony lesions of hydatid cysts are extremely mutilating and difficult to treat. Even with the best of efforts, they are associated with poor long-term functional outcomes with no possibility of rehabilitation [6,7].

We report, hereby, a case of a patient with a thoracic spine alveolar echinococcosis of an unusual extension.

## 2 Observation

A 62-year-old male, retired baker and former smoker, presented in January 2020 with spinal pain that he felt throughout the day. He described a throbbing pain, which has been developing for four months, which is worsened by movement. **He raised a dog in his teens** and he had a past medical history of a hydatid cyst of the left lung, operated on in 1978. After performing a cystectomy, it was no signs of recurrence till 2007. Medical investigation of persistent cough revealed the presence of a hydatic cyst of the dorsal spine. He had so, a spinolaminectomy from T6 to T9 in 2007. **Anatomopathological examination revealed a cyst wall within the bone trabeculae, formed by a double layer, internal corresponding to the nucleated proligeral membrane and a second layer located outside the previous one corresponding to the lamellar, anhistic cuticle**. Then, the patient remained asymptomatic for 13 years.

Clinical examination shows **a straightness of the dorsal rachis i.e. loss of normal kyphosis**.

A CT scan of the spine was done, showing a double convex thoracic scoliosis. Stigmata of stepped spinal laminectomy from T9-T11. Multiple thoracic vertebral corporeal osteolysis and T8-T10 vertebral block in relation to diffuse vertebral hydatidosis involving thoracic vertebrae with extension to adjacent soft tissues. There was a multifocal interruption of the posterior wall from T6 to T12. There were stigmata of a soft tissue cyst opening opposite T8-T9 in the left medio-basal sub-segmental bronchus and a mild right pleural reaction. There was no hepatic involvement. Hydatic serology was positive.

After a multi-disciplinary discussion, **the patient was put on an oral route Albendazole-based treatment for two years. We prescribed 400 mg twice daily, administered three days a week. Biological monitoring included serum ALAT and ASAT every four months. Enzyme assays were stable during follow-up.**

The thoracic surgeon decided to avoid a pulmonary surgical intervention, which would be a major risk of contamination.

A CT scan was performed after one year and showed stable bony and paravertebral involvement of the dorsal spine. It also showed a non-aerated collapse of the apical and posterobasal segments of the left inferior lobe – adjacent to the mediastinal pleura, with embedded air bubbles.

**Fig. 1:**
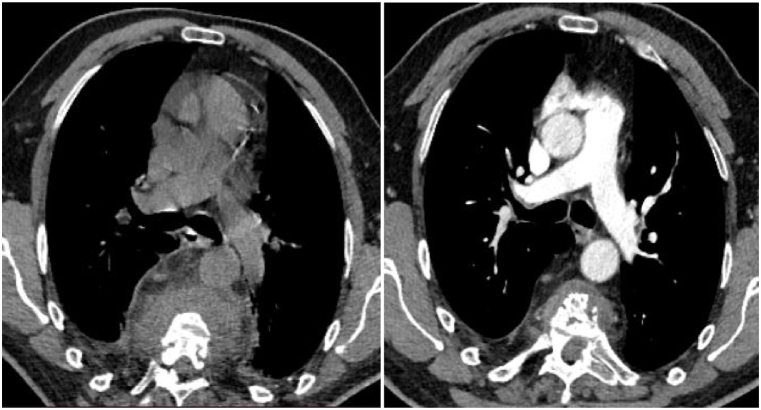
CT scan cross-section at T6 vertebra (2020 on the left and 2022 on the right).

**Fig. 2:**
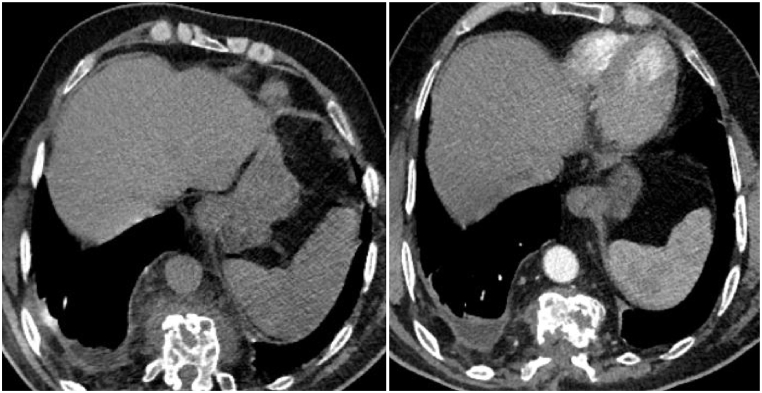
CT scan cross section at T11 vertebra (2020 on the left and 2022 on the right) showing a mild pleural effusion.

**Fig. 3:**
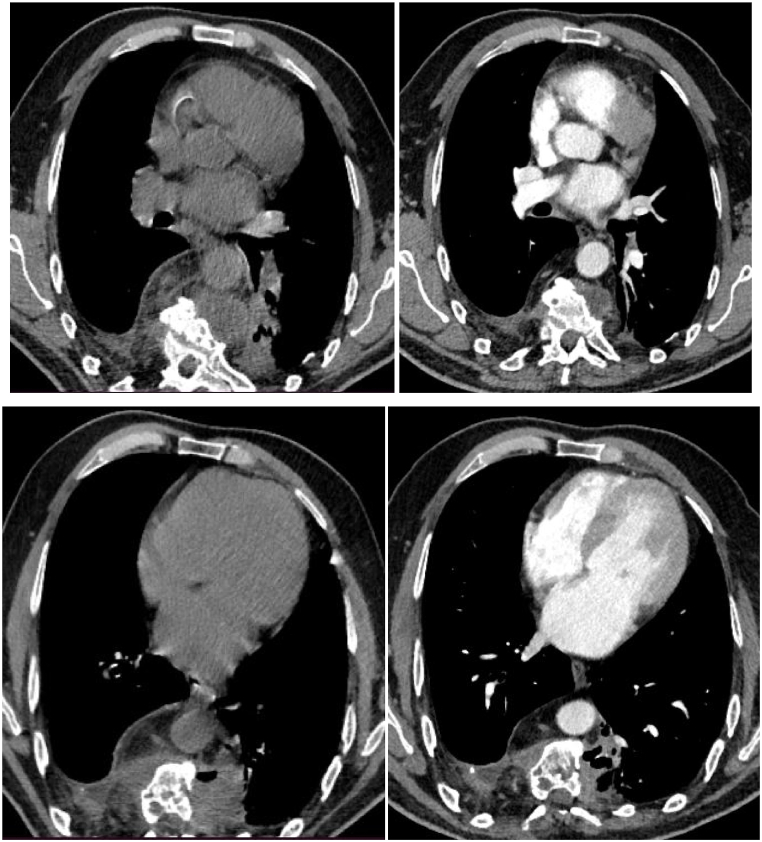
CT scan cross section at T8 vertebra and T9 vertebra (2020 on the left and 2022 on the right).

**Fig. 4:**
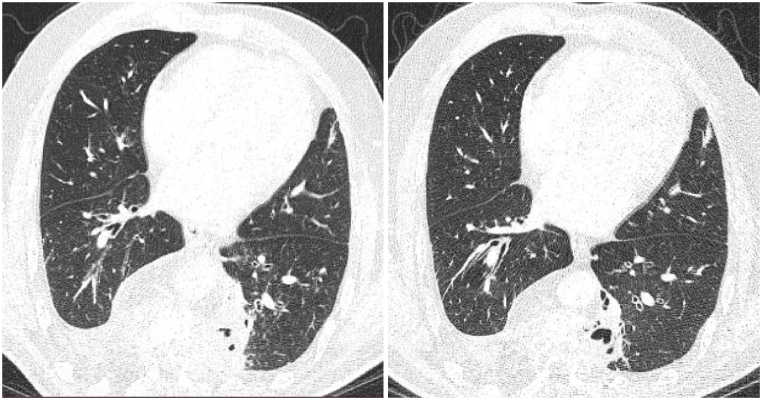
CT scan cross section at T9 vertebra with a parenchymal analysis (2020 on the left and 2022 on the right).

A follow-up thoracic scan two years after anti-helminthic treatment (Fig. 1, Fig. 2, Fig. 3, Fig. 4) showed a decrease in size of the paraspinal collections of the dorsal spine, stability of the bone involvement and of the right pleural collection, and non-aerated collapse of the apical and posterobasal segments of the left lung with small air collections.

These findings were interpreted as a good response to a well-tolerated treatment. In the meantime, the patient felt reduced back pain and quit using painkillers.

## 3 Discussion

The spine is the most common location of bone hydatidosis [4,5]. The diagnosis is difficult, and usually, the patients present with neural compression symptoms [6]. Radiographs show pedicle erosion and loss of vertebral body height. Myelography can be dangerous because the puncture of a cyst may lead to the intra-dural spreading of the disease and anaphylaxis. It only shows the blockage of the subarachnoid space, which is nonspecific. Preoperative aspiration of fluid is also hazardous because of the risk of dissemination or anaphylaxis. CT scanning and MR imaging techniques are indicated as the diagnostic procedures of choice [4,5]. A CT scan shows body, pedicle, and lamina erosions of the cancellous bone without a sub-periostal reaction. MRI is superior to CT scanning in evaluating the extent of the disease. It can give the full image of the vertebral axis of the spinal canal and thus reveals the full extent of the disease. It can help to plan the operation. It also can help evaluate recurrence.

Bone alveolar echinococcosis is a rare presentation, even more so without previous hepatic or pulmonary involvement. Our case is unique since we detected vertebral cystic echinococcosis before the pulmonary location. It is important to update our knowledge of all clinical presentations of this potentially disabling disease.

Trends over the past 20 years include increasing atypical manifestations and an epidemiological expansion of registered cases from rural towards urban areas [8,9].

In this paper, we report a case where the disease was well-controlled with medical therapy. Other researchers have reported cases where the disease was progressing slowly or relapsing after surgical removal of the cyst [10].

In hydatid disease of the vertebrae, the parasites spread along the bony intratrabecular space. The disease tends to propagate beneath the periosteum and ligaments, destroying the bone, so intervertebral discs are usually preserved. Neural compression is common, and spinal hydatidosis usually presents with paraplegia or nerve root compression [3,11,12].

PET-CT and serology are good tools for following up patients and can potentially be used to determine when to stop long-term medical treatment, but only in immunocompetent individuals [8,11,13].

Management entails surgical excision and at least 6 months of Albendazole therapy [14]. In the case of neural compression, surgical decompression and stabilization, combined with adjuvant Albendazole, is the treatment of choice. Our patient did not get the oral therapeutic course after his first spinal surgery in 2007.

Post-operative mortality is not rare. Morbidity and recurrence of the disease have high rates: 48% of those with vertebral disease have evidence of disease recurrence at 24 months. So, close follow-up with serial imaging is mandatory. Only 30–40% of patients with spinal hydatid cysts make a full recovery [5]. The multi-disciplinary decision to avoid further surgery in 2020 was wise. The cyst was difficult to separate from the adjacent structures. There was a major risk of echinococcal dispersion during surgery. Extension of the cyst to the lung created a natural fistulization through the bronchial tree. Follow-up has shown a rapid improvement, explained probably by a partial evacuation of the cyst.

Albendazole is the mainstay of drug therapy. Most practitioners prescribe it at a dose of 10–15 mg/kg per day divided into two daily doses [11,14]. Monitoring of hematopoiesis and liver enzymes is recommended during treatment, and pregnancy is a contraindication [15,16]. In extra-thoracic alveolar echinococcosis, indefinite medical treatment after incomplete surgery was associated with lower relapse rates than medical treatment for 2 years or less [11].

## 4 Conclusions

Bone echinococcosis is a rare parasitic disease. Authors are always defending a personalized approach taking account of the particularities of the cyst location. Although combined surgical and medical approaches are the standard of care, there is still risk of recurrence. Focus on the spinal hydatid cyst management is legitimate. Debate is still ongoing. Surgical procedures are challenging due to intimate proximity to the central nervous system and characteristics of the parasite, namely, its cystic structure and its capacity for expansion and invasion.

Our case is specifically interesting since it illustrates the potential risk of a late recurrence after surgical treatment and the efficacy of a medical protocol consisting of a minimized and well-tolerated Albendazole dose.

## Financial disclosure statement

This research did not receive any specific grant from funding agencies in the public, commercial, or not-for-profit sectors.

## Declaration of competing interest

Authors declare no conflicts of interest.
